# Genome-Wide Identification, Evolution, and Expression of GDSL-Type Esterase/Lipase Gene Family in Soybean

**DOI:** 10.3389/fpls.2020.00726

**Published:** 2020-06-25

**Authors:** Hong-Gang Su, Xiao-Hong Zhang, Ting-Ting Wang, Wen-Liang Wei, Yan-Xia Wang, Jun Chen, Yong-Bin Zhou, Ming Chen, You-Zhi Ma, Zhao-Shi Xu, Dong-Hong Min

**Affiliations:** ^1^College of Life Sciences, College of Agronomy, Northwest A&F University/State Key Laboratory of Crop Stress Biology for Arid Areas, Yangling, China; ^2^Institute of Crop Science, Chinese Academy of Agricultural Sciences (CAAS)/National Key Facility for Crop Gene Resources and Genetic Improvement, Key Laboratory of Biology and Genetic Improvement of Triticeae Crops, Ministry of Agriculture, Beijing, China; ^3^College of Agriculture, Yangtze University, Hubei Collaborative Innovation Center for Grain Industry, Engineering Research Center of Ecology and Agricultural Use of Wetland, Ministry of Education, Jingzhou, China; ^4^Shijiazhuang Academy of Agricultural and Forestry Sciences, Research Center of Wheat Engineering Technology of Hebei, Shijiazhuang, China

**Keywords:** GELP, expansion, intron gain and loss, gene duplication, expression profiles

## Abstract

GDSL-type esterase/lipase proteins (GELPs) belong to the SGNH hydrolase superfamily and contain a conserved GDSL motif at their N-terminus. GELPs are widely distributed in nature, from microbes to plants, and play crucial roles in growth and development, stress responses and pathogen defense. However, the identification and functional analysis of *GELP* genes are hardly explored in soybean. This study describes the identification of 194 *GELP* genes in the soybean genome and their phylogenetic classification into 11 subfamilies (A–K). *GmGELP* genes are disproportionally distributed on 20 soybean chromosomes. Large-scale WGD/segmental duplication events contribute greatly to the expansion of the soybean *GDSL* gene family. The Ka/Ks ratios of more than 70% of duplicated gene pairs ranged from 0.1–0.3, indicating that most *GmGELP* genes were under purifying selection pressure. Gene structure analysis indicate that more than 74% of *GmGELP* genes are interrupted by 4 introns and composed of 5 exons in their coding regions, and closer homologous genes in the phylogenetic tree often have similar exon-intron organization. Further statistics revealed that approximately 56% of subfamily K members contain more than 4 introns, and about 28% of subfamily I members consist of less than 4 introns. For this reason, the two subfamilies were used to simulate intron gain and loss events, respectively. Furthermore, a new model of intron position distribution was established in current study to explore whether the evolution of multi-gene families resulted from the diversity of gene structure. Finally, RNA-seq data were used to investigate the expression profiles of *GmGELP* gene under different tissues and multiple abiotic stress treatments. Subsequently, 7 stress-responsive *GmGELP* genes were selected to verify their expression levels by RT-qPCR, the results were consistent with RNA-seq data. Among 7 *GmGELP* genes, *GmGELP28* was selected for further study owing to clear responses to drought, salt and ABA treatments. Transgenic *Arabidopsis thaliana* and soybean plants showed drought and salt tolerant phenotype. Overexpression of *GmGELP28* resulted in the changes of several physiological indicators, which allowed plants to adapt adverse conditions. In all, *GmGELP28* is a potential candidate gene for improving the salinity and drought tolerance of soybean.

## Introduction

GDSL-type esterase/lipase protein (GELP, esterase, EC 3.1.1, lipase, EC 3.1.1) is a variety of hydrolytic enzyme (lipolytic enzyme) with broad substrate specificity, which can hydrolyze many kinds of substrates such as thioesters, aryl esters, phospholipids and amino acids (Akoh et al., 2004). GELPs hold unique structural features, possessing a conserved GDSL motif at their N-terminus, which is different from classic lipolytic enzymes containing the conserved motif GxSxG (Upton and Buckley, 1995). GDSL lipases are also named as SGNH hydrolases because of the four invariant important catalytic residues Ser, Gly, Asn and His present in conserved blocks (I, II, III and V), respectively. Meanwhile, the active-site serine of GDSL lipolytic enzymes (block I) as well as Asp and His residues (block V) composed a catalytic triad existing in all enzymes (Upton and Buckley, 1995; Akoh et al., 2004). Since first identified and reported the conserved domain PF00657 in bacteria, several studies have been conducted to research these fascinating lipolytic enzymes, and results demonstrate that GDSL lipases are spread widely among prokaryotes and eukaryotes (Chepyshko et al., 2012). To date, comprehensive studies have been carried out to systematically investigate the *GDSL* gene family in various species, and it was reported that there are 105, 114, 114, 96, 126 and 130 members of *GDSL* family in *Arabidopsis thaliana, Oryza sativa, Brassica rapa, Vitis vinifera, Populus trichocarpa* and *Sorghum bicolor*, respectively (Volokita et al., 2011; Chepyshko et al., 2012; Lai et al., 2017).

It has been demonstrated that *GDSL* family members play significant roles in regulating plant growth and development (Ma et al., 2018; An et al., 2019; Ding et al., 2019a; Watkins et al., 2019), organ morphogenesis (Smyth, 2017; Yadav et al., 2017; Zhang et al., 2017), secondary metabolism (Huang et al., 2015), plant immunity (Hong et al., 2008; Kwon et al., 2009; Lee et al., 2009; Kim et al., 2013, 2014; Rajarammohan et al., 2018; Ding et al., 2019b) and biotic and abiotic stresses (Naranjo et al., 2006; Hong et al., 2008; Kim et al., 2008). Very recently, it revealed that DARX1, a new active polysaccharide acetylesterase, regulating the conformation of arabinoxylan and the cross-linking mode with cell wall polymers of cellulose by acting on deacetylating the side chain of hemicellulose and arabinoxylan, thereby controlling the advanced structure and function of the cell wall. Not only that, DARX1 can also regulate the development of fiber cells and xylem vessels, and change the mechanical strength of rice (Zhang et al., 2019). AtCDEF1 (cuticle destructing factor 1), expressed specifically in mature pollen, which was demonstrated to be involve in the pollen-stigma interaction by degrading the cutin on the surface of the stigma in *Arabidopsis*. Beyond that, AtCDEF1 functions in facilitating the emergence of the lateral roots by degrading wall components (Takahashi et al., 2010). *Arabidopsis EXL4* (extracellular lipase 4) also may function in the pollen-stigma interface to facilitate hydration, which is required for efficient pollen hydration. *exl4*-*1* exhibited significantly reduced competitiveness in pollination and esterase activity compared with wild type (Updegraff et al., 2009). Similarly, *EXL6* genes perform a crucial role in pollen development in *Arabidopsis* and *Brassica rapa* L, and *BrEXL6* may be an ortholog of *AtEXL6* (Dong et al., 2016). It was reported that an endoplasmic reticulum-localized *GDSL* lipase, ZmMs30, specifically expressed in maize anthers, which is required for anther cuticle and pollen exine development (An et al., 2019). It was established that overexpression of *AtGDSL1* enhanced *Sclerotinia sclerotiorum* resistance in rapeseed by modulating SA- and JA-dependent pathways, resulting in increased accumulation of phosphatidic acid (PA) and activation of downstream stress response pathways after *Sclerotinia* infection (Ding et al., 2019b). *AtLTL1*, a novel halotolerance gene in *Arabidopsis*, was demonstrated to be involved in the response to salt stresses in yeast and transgenic plants (Naranjo et al., 2006).

Soybean, a member of the *Leguminosae* sp. family and native to China, is one of the most important global sources of seed protein and oil and rich in various beneficial nutrients, including isoflavones and vitamins. Genetically, soybean is an ancient palaeopolyploid and an outstanding model for the elucidation of the consequences of genome duplication in higher eukaryotes (Schlueter et al., 2007; Lee et al., 2013). Gene duplication can happen in varying degrees of completeness: whole genome duplication (WGD), segmental duplication or tandem repeat (Lyons and Freeling, 2008). WGD had resulted in a striking increase in several angiosperm lineages, such as Poaceae, Solanaceae and Fabaceae (Soltis et al., 2009). In 2010, the drafted genome sequencing of cultivar Williams 82 was completed, which set a basis to identify the soybean gene family at genome-level and also research the evolution of soybean genes in plants (Schmutz et al., 2010).

Although the *GDSL* gene family have been analyzed in several plant species, no systematical investigation are conducted in soybean. Given the significance of this gene family, an accurate genome-wide identification was performed in the current study. A comprehensive analysis of the *GDSL* gene family, including phylogenetic relationships, chromosomal location, gene duplication as well as expression profiles was performed to illuminate GELP’s evolutionary and functional characteristics. The insights gained from this study may be of assistance to better understand the extension and evolution patterns of *GELP* genes in the soybean genome, and highlight their function in regulating growth and development as well as abiotic stresses.

## Materials and Methods

### Plant Materials, Growth Conditions, and Stress Treatments

Soybean cultivar Zhonghuang 39 and Williams 82 were used to gene expression patterns and soybean hairy roots, respectively. *Arabidopsis* Col-0 plants were used for phenotypic assays. All the plants were grown in a greenhouse at 25°C with a photoperiod of 16-h light/8-h dark. One-week-old soybean seedlings were used for drought, salt and exogenous ABA treatments as previously described (Li B. et al., 2019). The leaves of seedlings under drought, salt and ABA treatments were collected at 0, 0.5, 1, 2, 4, 8, 12 and 24 h after treatments. All collected samples were frozen immediately in liquid nitrogen and then stored at −80C° for subsequent analysis.

### Identification of *GELP* Genes in Soybean

The identification of soybean *GDSL* gene family was performed according to the method described by Rao et al. with some revisions (Rao et al., 2010). Firstly, the soybean protein sequences were downloaded from the Phytozome database to build a local protein database (Goodstein et al., 2012). Then, the Hidden Markov model (HMM) profile of the GDSL conserved domain (PF00657) was used for the purpose of scanning the local database using the local BLASTP program (with *E*-value < e^–20^), and then all obtained sequences were aligned and used to build a soybean-specific HMM profile of GDSL domain using the hmmbuild program from the HMMER v3 (Finn et al., 2011). Then, the new soybean-specific HMM profile was used to search for GELP members from the local protein database using hmmsearch program. All candidate proteins were submitted to NCBI Batch CD-search and SMART databases for examining the presence of the GDSL conserved domain (Marchler-Bauer et al., 2015). After validation, all candidate genes encoding the GDSL domain were identified in the soybean genome (Supplementary Table S1).

### Multiple Sequence Alignment and Phylogenetic Analysis

Multiple sequence alignment was performed using MAFFT software with the E-INS-I option version 7. Non-aligned regions were removed with Gblocks version 0.91b. LG+I+G, LG+I+G and JTT+G models with a 4-categories GAMMA distribution were used in the phylogenetic trees of *Arabidopsis* and soybean, subfamily I and subfamily K GELP proteins, respectively, they were identified using ProTest. Possion model and pairwise deletion was used in the phylogenetic tree of soybean GELP proteins. All the phylogenetic trees were reconstructed using MEGA version 7. A bootstrap test with 1000 replications was used to determine the statistical reliability of the phylogenetic trees. The similarities of eight reported AtGELP proteins and each corresponding homologous protein in soybean are shown in Supplementary Table S2.

### Chromosomal Location and Gene Duplication

All *GELP* genes were mapped on the 20 chromosomes of soybean using positional information acquired from the phytozome database by TBtools software (Chen et al., 2018). Distribution frequency of *GELP* genes on 20 soybean chromosomes was shown in Supplementary Figure S1. Segmental and tandem duplication events were determined as previously described (Wang et al., 2016; Fan et al., 2019). Briefly, a pair of duplicated *GmGELP* genes were defined when the alignments covered > 80% of the longer gene and the aligned region had an identity > 80% at the nucleotide level. Tandem duplication event was demarcated based on the chromosomal location of each of duplicated gene. Related synteny blocks and duplicated gene pairs in soybean were obtained and visualized using TBtools software. The nonsynonymous substitution rate (Ka), synonymous substitution rate (Ks) and the Ka/Ks ratio between paralogous gene pairs were evaluated using a comparative synteny map within the soybean genome (*Glycine max* Wm82.a2.v1) by TBtools software, and detailed information of duplicated gene pairs can be found in Supplementary Table S3.

### Gene Structure, Intron Pattern and Conserved Motifs

The gene structure information and intron insertion sites of *GmGELP* genes were obtained from the phytozome database and visualized using the GSDS (Gene Structure Display Server) program and DNAMAN software, respectively (Hu et al., 2015). The schematic of conserved motifs of all members is presented in Supplementary Figure S2 based on the analysis of online MEME program, and the maximum number of motifs was set to twenty (Bailey et al., 2009).

### Expression Pattern Detected by Transcriptome Data

To study the expression of *GmGELP* genes in different tissues and organs, transcriptome data extracted from a public soybean database were used to investigate the different expression of *GmGELP* (Severin et al., 2010; Du et al., 2018). RNA-seq data of various abiotic stresses were extracted from our previous research to study the expression of *GELP* genes under salt (4 h), drought (4 h) and ABA treatments (3 h) (Shi et al., 2018). FPKM (fragments per kilobase of transcript per million mapped reads) values of soybean *GELP* genes in different tissues and under abiotic stress treatments (drought, NaCl and ABA) were shown in Supplementary Tables S4–S7. A heatmap showing tissue-specific expression profiles was generated using the log2-transformed (FPKM + 1) values of *GmGELP* genes, and the expression level fold change realtive to the normal control was used to heatmaps for abiotic stress conditions. Moreover, differentially expressed *GmGELP* genes with significant level (fold-change ≥ 2 and *p*-value ≤ 0.01) were used to analyze the expression of *GmGELP* genes under three abiotic stresses. Finally, visualization of the expression levels of *GmGELP* genes was accomplished using TBtools software.

### RNA Extraction and RT-qPCR

Total RNA was isolated from soybean leaves subjected to the multiple stress treatments using RNA plant extraction kit (Zhuangmeng, Beijing, China) according to the manufacturer’s instruction. Approximately 2 μg of purified total RNA from each sample was used in reverse transcription using TransScript One-Step gDNA Removal and cDNA Synthesis SuperMix (TransGen Biotech, Beijing, China) and stored at −20°C. RT-qPCR analysis was accomplished using PerfectStart Green qPCR SuperMix (TransGen Biotech, Beijing, China) and an Applied Biosystems 7500 Real-Time PCR System (ThermoFisher, Beijing, China). Data analysis was conducted using the 2^–ΔΔCT^ method. Three technical replicates were performed for each of the three biological replicates. The primers used for RT-qPCR in this study were given in Supplementary Table S8.

### Identification of *Cis*-Elements in the Promoters of 7 *GmGELP* Genes

For *cis*-elements analysis, 2 kb sequences upstream from the start codons of 7 *GmGELP* genes were downloaded from the Phytozome database and analyzed using the PlantCARE database.

### Generation of Transgenic *Arabidopsis* Plants

The *GmGELP28* coding region was amplified and inserted into the pCAMBIA1302 vector under the control of the CaMV35S promoter. The resulting construct pCAMBIA1302-GmGELP28 was transformed into *Arabidopsis* Col-0 plants using the floral dip method. The harvested seeds were surface sterilized with sodium hypochlorite and germinated on 1/2-strength MS media. Three *GmGELP28* transgenic lines were selected for further study.

### *Agrobacterium rhizogenes*-Mediated Transformation of Soybean Hairy Roots

To generate the pCAMBIA3301-GmGELP28 overexpression vector, the coding region of *GmGELP28* was amplified from Williams 82 cDNA, and the PCR product was then ligated into the pCAMBIA3301 vector under the control of the CaMV35S promoter. The recombinant vector was transfermed into soybean hairy roots by *Agrobacterium rhizogenes*-mediated transformation following the protocol described previously (Kereszt et al., 2007; Su et al., 2019). After verification, positive soybean hair roots were used to abiotic tolerance assays, and 8 plants per pot were used in three to six biological replicates.

### The Abiotic Stress Responses of Transgenic *Arabidopsis* Plants and Soybean Hairy Roots

For drought tolerance assays, 3-week-old *Arabidopsis* plants in soil were subjected to drought treatment by halting watering. The plants were photographed when differences in phenotype were observed. Watering was reinitiated after 2 weeks for plant growth recovery. About a week later, the plants were photographed again and calculated the survival rates. In addition, salt stress was applied by the addition of 250 mM NaCl solution to the soil-grown *Arabidopsis* plants for 1 week, and control plants were grown under normal conditions.

Soybean drought tolerance assays were run as described above. In brief, 2-week-old soybean plants with transgenic hairy roots were subjected to dehydration for 2 weeks and rewatered for 3 days. With respect to salt treatment, 2-week-old soybean plants with transgenic hairy roots were treated with 200 mM NaCl solution for 3 days.

### The Quantification of MDA, Proline, Chlorophyll and H_2_O_2_

The contents of malonaldehyde (MDA), Proline, chlorophyll and H_2_O_2_ were detected according to the instructions of the corresponding measurement kit (Cominbio, Suzhou, China). Three replicates were included per measurement. The leaves in each pot were mixed and sampled. The contents of MDA, Proline and chlorophyll of *Arabidopsis* samples were detected after drought treatment for 2 weeks and salt treatment for 1 week. As to soybean samples, the contents of MDA, Proline and H_2_O_2_ were detected after drought treatment for 10 days and salt treatment for 2 days when the leaves were slightly wilted. Each experiment was performed in triplicate.

## Results

### Identification of the *GELP* Genes in Soybean

A total of 194 candidate genes encoding the GDSL domain were identified in the soybean genome (*Glycine max* Wm82.a2.v1). They were named as *GmGELP1*-*GmGELP194* according to their chromosomal locations. Detailed information about these predicted genes is summarized in Supplementary Table S1. The protein lengths of coding sequences range from 266 (GmGELP90) to 460 amino acids (GmGELP77), with the average sequence length of 346 amino acids. The lengths of *GmGELP* genes in genome vary from 974 to 14351 bp.

### Phylogenetic Analysis of *GmGELP* Genes

**FIGURE 1:**
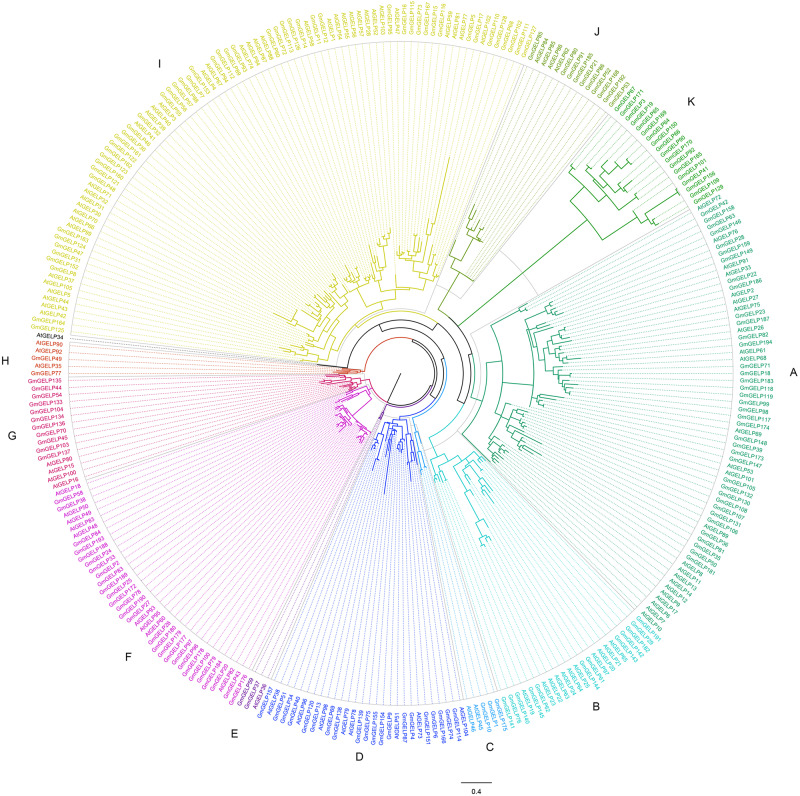
The phylogenetic analysis of GELP proteins in soybean and *Arabidopsis*. The branches of different subfamilies are marked using different colors.

As described in a previous study (Lai et al., 2017), to better elucidate the phylogenetic relationship among *Arabidopsis* and soybean *GELP* genes, the homology sites of 194 GmGELP and 104 AtGELP proteins (except AtGELP29, which is absent from *Arabidopsis*) were used to produce a phylogenetic comparative tree (Figure 1). Combining MEME analysis and intron number statistics, 298 GELP members were further divided into 11 subfamilies containing 3 to 87 members each. Confusingly, *AtGELP34* is not clustered with any of the other members. In most of these subfamilies, the same subfamily also contains soybean *GELP* genes and *Arabidopsis GELP* genes, suggesting possible conservation of function within dicot species. However, all 18 members of subfamily K are from soybean, implying that *GmGELP* genes in subfamily K might have occurred very early, before the divergency of soybean and *Arabidopsis*, which may be potential candidate genes to distinguish soybean and *Arabidopsis.* Among the 8 AtGELP proteins (AtESM1, AtGLIP1, AtGLIP2, EXL4, CDEF1, SFAR1-5, LAE and AtLTL1) whose functions had been reported, 6 genes were found to share more than 38% similarity with their homologous proteins, and gathered together in the same subfamily of phylogenetic tree (Supplementary Table S2). However, it’s worth noting that there is no clear association between biological function and specific clades of phylogenetic tree.

### Chromosomal Location, Motif Identification, and Duplication Events Analysis

**FIGURE 2:**
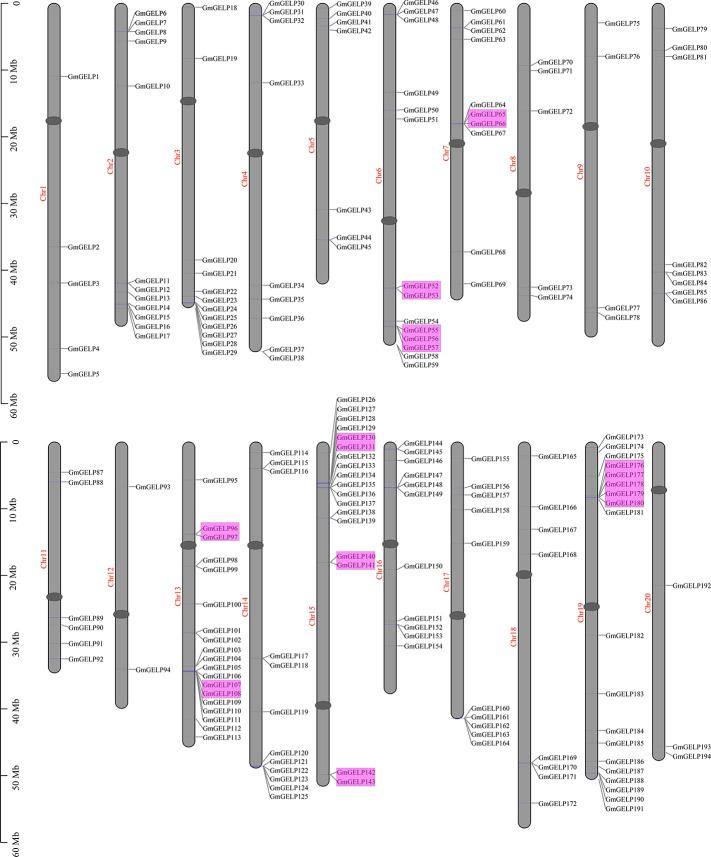
Chromosomal locations of the 194 *GmGELP* genes. The tandemly duplicated genes are labeled in purple boxes. The chromosome numbers are marked left of each chromosome, centromeric regions are indicated by ellipses. The bar located on the left side shows the size of chromosome in megabases.

As can be seen from Figure 2 and Supplementary Figure S1, 194 *GmGELP* genes are universally and unevenly distributed on all 20 soybean chromosomes, similarly, to the distribution characteristics of *GELP* genes in the rice and *Arabidopsis* genomes found in previous studies (Dong et al., 2016; Lai et al., 2017). Three chromosomes contain approximately 29% (56 out of 194) of *GmGELP* genes: chromosome 13 (19 genes, 9.8%), chromosome 15 (18 genes, 9.3%) and chromosome 19 (19 genes, 9.8%), whereas only 5, 5, 4, 2 and 3 *GmGELP* genes are located on chromosomes 1, 8, 9, 12 and 20, respectively. Unsurprisingly, the majority of *GmGELP* genes are located on chromosome ends, which is confirmed by a prior report describing that about 78% of the predicted genes are located on chromosomal ends (Schmutz et al., 2010).

**FIGURE 3:**
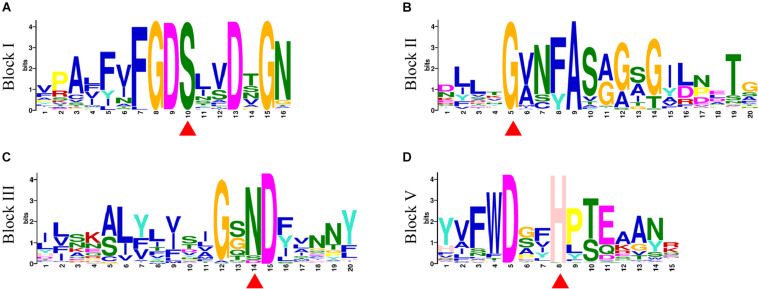
**(A–D)** Motif logos of four conservative blocks (Block I, II, III and V) detected in GmGELP proteins via MEME analysis, respectively. The names of motifs are listed on the left. Conservative amino acid residues Ser-Gly-Asn-His in blocks are marked by red triangles.

A total of 20 conserved motifs were discovered from 198 GmGELP proteins (with *E*-value below 9.40E-159) and displayed in Supplementary Figure S2. Motifs 1, 4, 5 and 8 represent the conserved blocks I, II, III and V of GDSL family, respectively, which are present in almost all 198 proteins (Figure 3). In addition, other 6 well-conserved motifs (motifs 2, 3, 6, 9, 10 and 12) were detected in more than 60% of GmGELP proteins (Supplementary Figures S2, S3). However, several motifs are specific to individual subfamilies in the phylogenetic tree (Supplementary Figure S2). For instance, subfamily F possesses specific motifs 16 and 18, while motifs 17,19 and 20 are only detected in subfamily K. These results indicate that GmGELP members of the same subfamily often have similar motif composition, which is consistent with their phylogenetic relationship.

**FIGURE 4:**
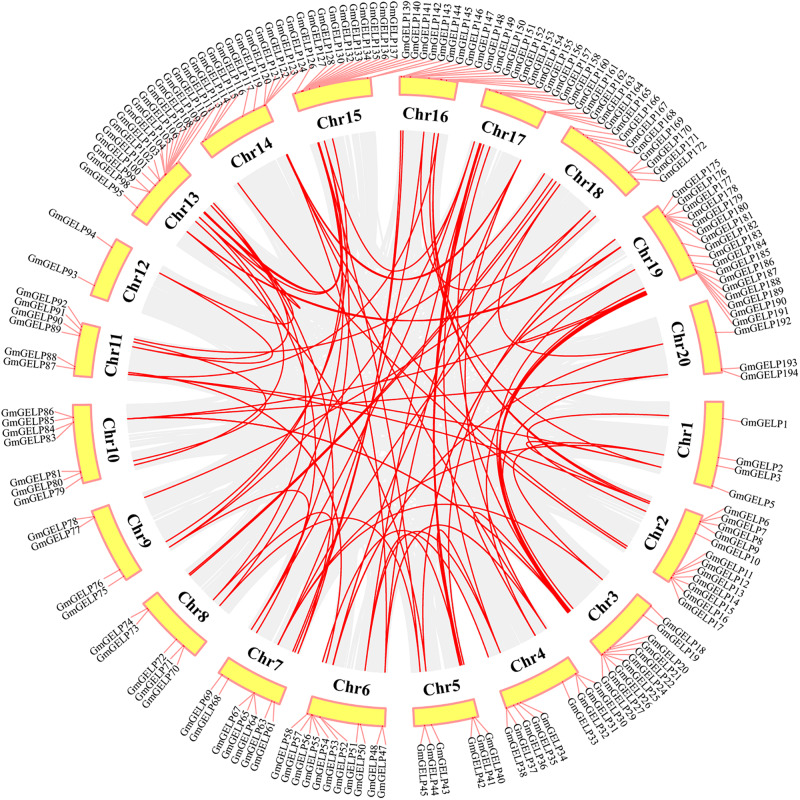
Distribution and WGD/segmental duplication of *GELP* genes on soybean chromosomes. The panel shows the 20 soybean chromosomes in a circle with red lines connecting homologous genes, gray regions indicate all synteny blocks within the soybean genome, and the chromosome numbers are indicated at the inside of the circle.

To elucidate the amplification mechanism of *GmGELPs*, we conducted collinearity alignment within the soybean genome, indicating that a total of 139 *GmGELP* genes are located within syntenic blocks on soybean chromosomes (Figure 4 and Supplementary Table S3). Tandem duplication is defined according to the methods previously reported (Holub, 2001; Li Z. et al., 2019). Statistical results showed that approximately 11% (22 out of 194) of *GmGELP* genes were found to derive from tandem duplication events (Supplementary Table S3), and 9 tandemly duplicated *GmGELPs* sets contain 2-5 *GmGELP* genes. Among them, 7 tandemly duplicated *GmGELP* sets contain 2 members (*GmGELP52*/*53*, *GmGELP6*/*66*, *GmGELP96*/*97*, *GmGELP107*/*108*, *GmGELP130*/*131*, *GmGELP140*/*141* and *GmGELP142*/*143*), 1 tandemly duplicated *GmGELP* set contain 3 members (*GmGELP55*, *56* and *57*), and 1 tandemly duplicated *GmGELP* set is involved in 5 members (*GmGELP176*, *177*, *178*, *179* and *180*). Beside tandem duplication events, we further observed that up to 71% (137 out of 194) of *GmGELP* genes had participated in WGD/segmental duplication, which is much higher than the 21% in *Arabidopsis* genome (22 out of 105). In summary, these results suggest that WGD/segmental duplication is the main driving force for the large expansion of *GELP* genes in the soybean genome.

**FIGURE 5:**
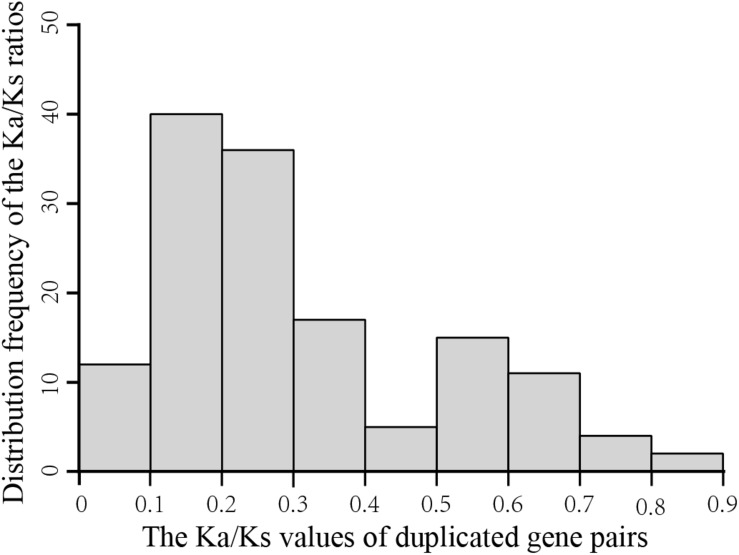
Histogram of distribution frequency of pairwise Ka/Ks ratios for homologous genes.

To explore the evolutionary forces acting on the 198 *GmGELP* genes, we evaluated the non-synonymous/synonymous substitution ratio (Ka/Ks) for each duplicated gene pair (Supplementary Table S3). The results suggest that the Ka/Ks ratios of *GmGELP* gene pairs are commonly less than 1 and range from 0.08 to 1.66 with an average of 0.30. Figure 5 shows an overview of the distribution frequency of the Ka/Ks ratios, results found that the Ka/Ks ratios of more than 70% of duplicated gene pairs ranged from 0.1 to 0.3, indicating that these duplicated *GmGELP* genes were under purifying selection pressure.

### Intron Loss and Gain Events and Gene Structure

The exon-intron structure, intron size and intron position showed high conservation among soybean, which can be used to derive phylogenetic relationship (Fedorov et al., 2002; Babenko et al., 2004; Roy and Penny, 2007). A striking feature of plant *GELP* genes is their structure of five exons and four introns. For instance, approximately 67.6% and 49.1% of *GELP* members contain 4 introns in *Arabidopsis* and rice, respectively (Ling et al., 2006; Chepyshko et al., 2012). The intron numbers of soybean *GELP* genes were calculated in our study (Supplementary Figure S4). The results demonstrate that intron numbers vary from 1 to 6, and up to 74.2% (143 out of 194) of *GmGELP* genes harbor 4 introns. All genes contain at least 1 intron, and the highest number of introns exist in the *GmGELP112* gene (7 exons and 6 introns) (Supplementary Table S1). We further analyzed the number of introns in each subfamily (Table 1). What stands out in Table 1 is that up to 56% (10 out of 18) genes of subfamily K possess 5 introns, and about 28% (14 out of 50) genes of subfamily I contain less than 4 introns. For this reason, subfamilies K and I were used to simulate the events of intron gain and loss, respectively.

**TABLE 1:** Intron numbers in each subfamily of *GmGELP* gene family.

Subfamily	1 Intron	2 Introns	3 Introns	4 Introns	5 Introns	6 Introns	Total
A	2	3	1	29	3	0	38
B	0	0	0	11	1	0	12
C	0	0	0	3	0	0	3
D	0	0	0	18	2	0	20
E	0	0	0	1	1	0	2
F	0	0	1	27	0	0	28
G	0	0	0	11	0	0	11
H	0	0	0	2	0	0	2
I	0	12	2	33	2	1	50
J	4	0	4	2	0	0	10
K	0	1	0	7	10	0	18
Total	6	16	8	144	19	1	194

**FIGURE 6:**
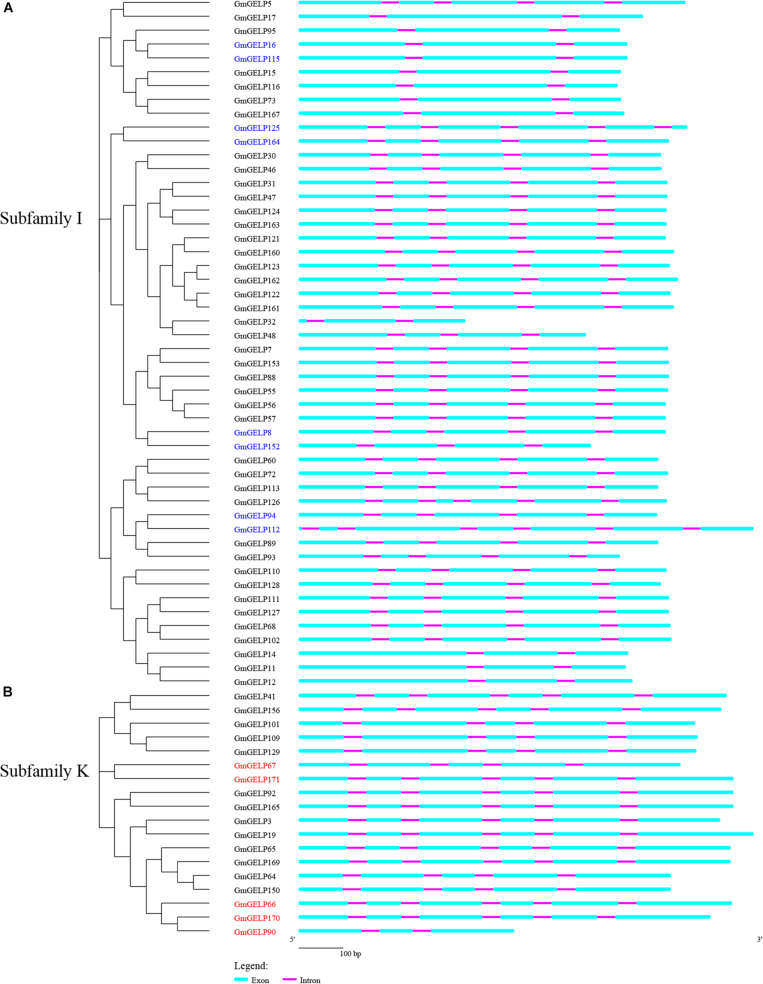
**(A,B)** Phylogenetic tree and gene structure analysis of *GmGELPs* from subfamilies I and K, respectively. Exon and intron are indicated by azure round-cornered rectangle and purple rectangle, respectively. Exon lengths are displayed proportionally and introns are scaled to the same length. 8 and 5 subfamily I and K genes for further study are marked with blue and red, respectively.

Prior studies showed that evolution of multi-gene families resulted in gene structure diversity (Luo et al., 2018). The exon-intron organization of 50 and 18 *GmGELP* genes from subfamilies K and I are shown in Figures 6A,B, respectively. Generally, adjacent members of the same branch have similar arrangements in terms of intron number and exon length, suggesting that exon-intron structure and phylogenetic tree are highly correlated. However, a minority of homologous gene pairs, for example, *GmGELP94/112*, presents slight differences in intron number and exon length. In fact, structural divergences have been widespread in duplicated genes, which could generate functionally distinct paralogs, whereas it is still unclear the mechanism that how the change of gene structure have promoted the generation of functionally distinct paralogs (Xu et al., 2012).

**FIGURE 7:**
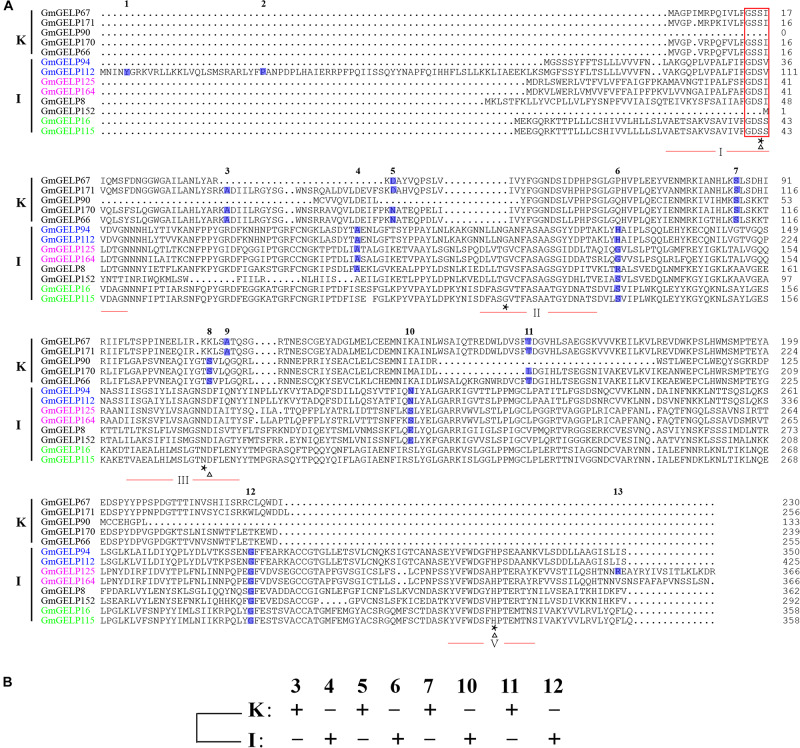
Different gene structures and later events of intron gain and loss. **(A)** Different gene structures represent the phylogenetic subfamilies. Multiple sequence alignment of the amino acid sequence of two types of GELP representatives from subfamilies K and I. The core short signature is marked by red rectangle. The four conserved blocks (I, II, III and V) of GELP proteins are underlined in red. Triangle and asterisk are used to indicate the residues of catalytic triad and four invariant important catalytic residues Ser-Gly-Asn-His in conserved blocks, respectively. The positions and numbers of introns are indicated by blue boxes and numbers above the boxes. 13 introns were denoted as 1 to 13 according to their relative position to the coding DNA strand. Homologous genes pairs are marked in the same colors. **(B)** A new model for revealing ancestral gene structure based on 8 conserved intron positions of subfamilies K and I, namely intron positions 3, 4, 5, 6, 7, 10, 11 and 12.

To further explain the reason for the variation in intron numbers of 68 genes in subfamilies K and I, 8 subfamily I and 5 subfamily K *GmGELP* genes were selected for analysis of intron position. Among them, three pairs of duplicated genes were included among them: *GmGELP94/112*, *GmGELP125*/*164* and *GmGELP16*/*115*. The intron positions of all 13 selected genes were identified in Figure 7A. Unsurprisingly, 13 selected *GmGELP* genes show a high degree of similarity, especially for the four invariant key Ser, Gly, Asn and His residues in the catalytic sites. However, none of the 13 GmGELP proteins contain the GDSL motif in N-terminal, and two lack the core short signature, indicating the sequence GDSL seems to be relatively conservative among GmGELP proteins, which had been confirmed by previous reports that GDSL/V motif existed in several GELP proteins of *Arabidopsis* and *Tanacetum cinerariifolium (Gao et al., 2017).* Four intron gene structure was fixated as the basic form in prior study (Volokita et al., 2011). According to the method just described, the intron positions 3, 5, 7, 11 and 4, 6, 10, 12 were defined as the basic forms of subfamilies K and I in our study. As can be seen from Figure 7A, intron positions are similar within intra-subfamily, but totally different between subfamilies K and I. Among three duplicated gene pairs, *GmGELP16* and *GmGELP115* show similar intron positions and contain only 2 introns, while *GmGELP94* and *GmGELP112* display different intron characteristics. Specifically, *GmGELP94* and *GmGELP112* contain 4 shared intron positions (the intron positions 4, 6, 10 and 12), while the intron positions 1 and 2 are unique to *GmGELP112*. Similarly, the intron position 13 exists only in *GmGELP125*. Based on the results of the above analysis, we speculate that the intron positions 1, 2 and 13 are produced by independent intron gain events, since extra introns exist in only a few genes. Conversely, the intron positions 4 and 10 are absent in *GmGELP16* and *GmGELP115*, which supports the hypothesis that reduced introns are the result of later intron loss events. Moreover, we consider these events occur frequently in several genes of subfamilies K and I.

Furthermore, to reveal the ancestral gene structure of subfamilies K and I, a new model was constructed based on the 8 conserved intron positions (3, 4, 5, 6, 7, 10, 11 and 12) (Figure 7B). We presume that the differences in intron position mainly resulted from gene structure diversity, or, alternatively, from events of intron loss and gain.

### Gene Expression Patterns Analysis

**FIGURE 8:**
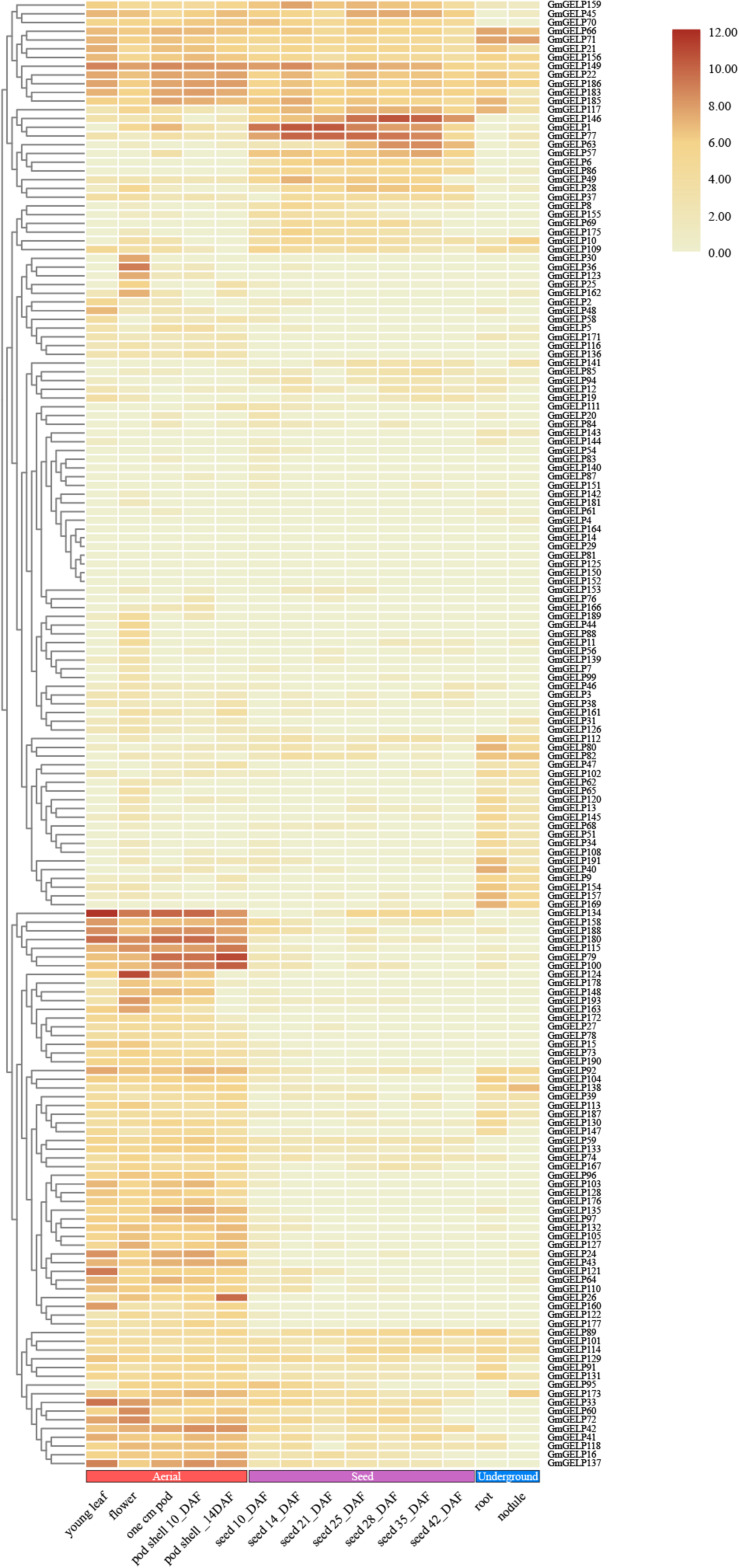
Hierarchical clustering of expression profiles of 168 available *GmGELP* genes in 14 different stages or tissues of soybean. Aerial, seed and underground tissues are represented in color for each boxed as red, purple and blue, respectively. The color intensity represents the degree of expression, as shown in the bar at top of figure.

The RNA-seq data of *GmGELP* genes in different tissues and development stages can give reference to molecular mechanisms of plant growth and development (Severin et al., 2010). The transcription levels of a total of 168 available genes were presented in Figure 8. The expression levels of 7 genes, namely *GmGELP14*, *29*, *81*, *125*, *133*, *152* and *164*, were not detectable, which led us to speculate that they were not expressed in the examined tissue/stage/condition, or that they were pseudogenes. A considerable number of members showed very low or no transcriptional abundance, including *GmGELP4*, *7*, *20*, *54*, *61*, *76*, *83*, *87*, *140*, *142*, *143*, *144*, *151* and *181*. However, a small portion of members were expressed constitutionally in soybean. For instance, *GmGELP22*, *GmGELP149* and *GmGELP186* exhibited high transcription levels in most tissues and organs throughout the growth period. Meanwhile, large amounts of genes showed tissue-specific expression, such as *GmGELP10*, *79*, *115*, *124* and *180* showed preferential expression in young leaves, flowers, pods and pod shells, while being expressed at low levels in roots and nodules, suggesting they may play distinct roles in special tissues or developmental stages. To explore the functional redundancy and differentiation of soybean *GELP* homologous gene pairs, the expression patterns of several duplicated genes were also investigated (Figure 8). Results indicated that some duplicated gene pairs showed similar expression patterns (for instance, *GmGELP21*/*185*, *GmGELP22*/*186*, *GmGELP24*/*188*, *GmGELP110*/*128* and *GmGELP34*/*40*/*51*/*157*). Whereas some homologous genes exhibited completely different or converse expression patterns (such as *GmGELP65*/*66*, *GmGELP70*/*137*, *GmGELP87*/*154*, *GmGELP109*/*129* and *GmGELP111*/*127*), suggesting that they may have undergone functional difference. In addition, we examined the expression level of *GmGELP20*, a homologous gene of *Arabidopsis GLP1*, indicating *GmGELP20* was expressed at extremely low levels in all tissues, while *AtGLP1* showed a higher transcript levels in seedlings, stems and roots (Kwon et al., 2009). In turn, *GLP2*, which was highly homologous with *Arabidopsis GLP1*, was expressed only in seedlings, roots and stems (Lee et al., 2009), while its homologous gene *GELP62* in soybean had extraordinarily weak to no expression abundance in all tissues (Lee et al., 2009). The overall expression data analysis indicated that *GmGELP* genes exhibited great disparities in abundance among different tissues, which might play significant effects in accommodating different physiological processes.

**FIGURE 9:**
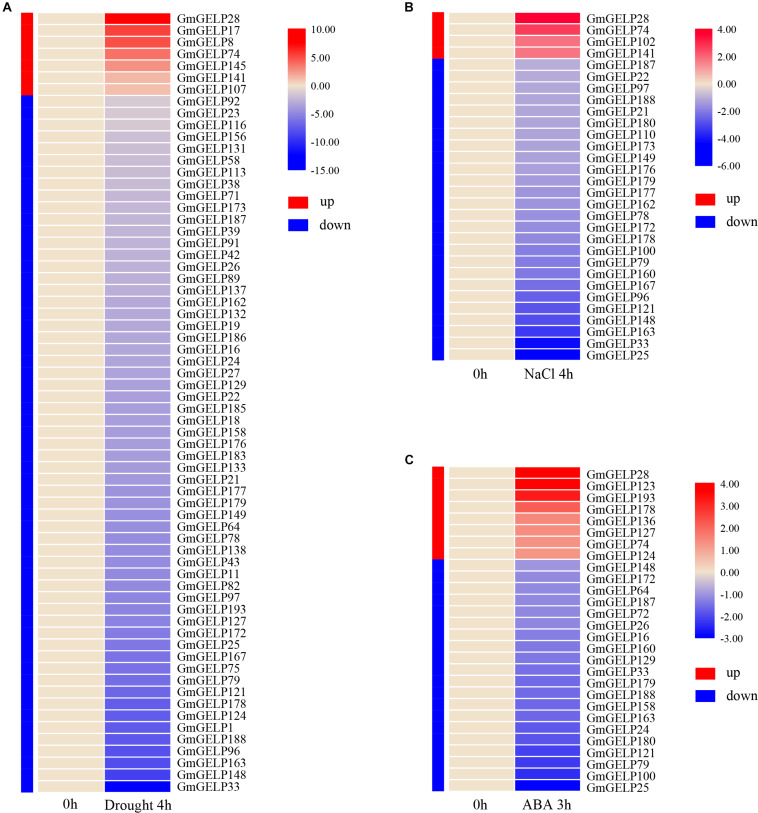
**(A–C)** Hierarchical clustering of expression profiles of 66, 30, and 28 drought-, NaCl- as well as ABA-responsive genes, respectively. Red and blue represent up-regulated and down-regulated expression, respectively. The color intensity indicates the expression level fold change compared to the normal control.

Extensive studies have shown that the expression of multiple *GDSL* family members can be induced by various abiotic stresses in some species (Naranjo et al., 2006; Hong et al., 2008; Huang et al., 2015; Li et al., 2017). To reveal the expression of *GmGELP* genes in response to abiotic stress, the relative expression abundances of all *GmGELP* genes in young leaves were investigated under three abiotic stress conditions (drought, salt and ABA). RNA-seq data indicated a total of 66, 30 and 28 *GmGELP* genes responded to drought, NaCl and ABA treatments, respectively (Figure 9). Overall, 89.4% (59 out of 66), 86.7% (26 out of 30) and 71.4% (20 out of 28) *GmGELP* genes were down-regulated under the conditions of drought, NaCl and exogenous ABA treatments, respectively. Meanwhile only 10.6% (7 out of 66), 13.3% (4 out of 30) and 28.6% (8 out of 28) *GmGELP* genes were up-regulated under the same conditions. Among them, several genes, such as *GmGELP1*, *GmGELP75*, *GmGELP11*, *GmGELP43* and *GmGELP8* were specifically expressed under drought stress, *GmGELP17*, *GmGELP102* and *GmGELP110* were specifically expressed under NaCl stress, as well as *GmGELP123* and *GmGELP136* were significantly up-regulated under exogenous ABA treatment (Supplementary Tables S5–S7). Another important finding is that 10 *GmGELP* genes were down-regulated under all three types of abiotic stresses, including *GmGELP25*, *GmGELP33*, *GmGELP79, GmGELP121*, *GmGELP148*, *GmGELP163*, *GmGELP172*, *GmGELP179*, *GmGELP187* and *GmGELP188*, whereas *GmGELP28* and *GmGELP74* were up-regulated under the same conditions. Interestingly, most homologous gene pairs showed a similar expression pattern under NaCl and drought stresses such as *GmGELP33*/*188*, *GmGELP78*/*172* and *GmGELP97*/*179*, indicating that they might perform similar physiological functions. Nevertheless, several duplicated gene pairs displayed different expression patterns. For example, *GmGELP178* was significantly up-regulated after ABA treatment, while *GmGELP179* was down-regulated, suggesting there may have arisen functional difference in these genes.

**FIGURE 10:**
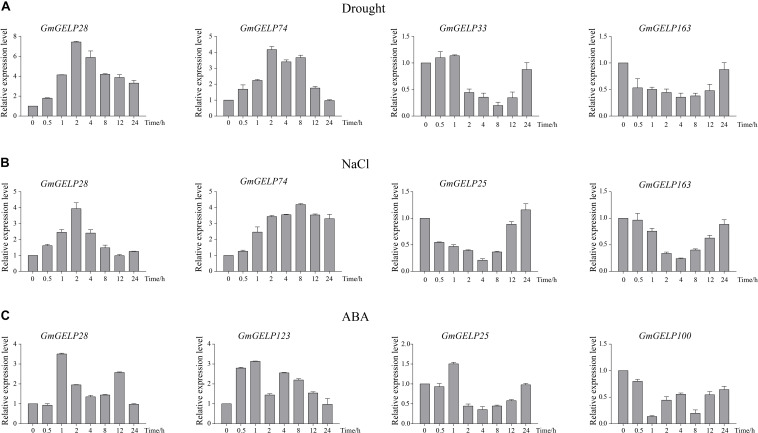
**(A–C)** Hierarchical clustering of expression profiles of 66, 30, and 28 **(A–C)** RT-qPCR analysis of 7 soybean *GmGELP* genes under abiotic stress treatments (drought, NaCl and ABA, respectively). The *actin* gene was used as an internal control. The mean expression value was calculated from three biological replicates.

Based on the results of RNA-seq, we observed that several genes were significantly induced by abiotic stresses. For this reason, 7 *GmGELP* genes were selected to confirm their expression patterns by RT-qPCR (Figure 10). Unsurprisingly, RT-qPCR results were consistent with RNA-seq data. For instance, *GmGELP28* showed a similar expression pattern under three abiotic stress treatments. Under drought stress, *GmGELP163* showed a slightly down-regulated expression (Figure 10A). *GmGELP33* and *GmGELP163* showed remarkable down-regulation after NaCl treatment (Figure 10B). Both *GmGELP28* and *GmGELP123* were significantly up-regulated at 1 h after ABA treatment (Figure 10C). The expression profiles of these stress-induced *GmGELP* genes provide valuable information for further revealing their roles under different abiotic stresses.

The *cis*-elements in promoter regions play important roles in regulating gene transcription and abiotic stress responses. Therefore, 2 kb sequences upstream from the start codons of 7 *GmGELP* genes were downloaded and analyzed using the PlantCARE database. The number of 6 abiotic stress response elements were counted and displayed in Table 2, including ABA-responsive elements (ABRE), drought-inducible elements (MBS, MYB binding site), low-temperature responsive elements (LTR), MEJA-responsive elements (CGTCA-motif), SA-responsive elements (TCA-element) and defense and stress responsive elements (TC-rich repeats). Among them, ABRE, MBS and CGTCA-motif were detected in almost every promoter region of the *GmGELP* genes. Note, the correlation between *cis*-elements and responses to abiotic stresses of genes needs further experimental validation. Nevertheless, *cis*-element analysis indicated that *GmGELP* genes might respond abiotic stresses.

**TABLE 2:** Number of variations of *cis*-elements in the promoter region of 6 *GmGELP* genes.

Gene	ABRE	MBS	LTR	CGTCA-motif	TCA-element	TC-rich repeats
GmGELP25	2	4	0	2	1	0
GmGELP28	1	1	0	3	1	1
GmGELP33	1	0	5	1	3	0
GmGELP74	2	2	0	0	0	3
GmGELP100	4	1	0	1	0	1
GmGELP123	2	1	0	1	1	0
GmGELP163	0	2	0	2	1	0
Total	12	11	5	10	7	5

### *Arabidopsis* Plants Expressing *GmGELP28* Were More Tolerant of Drought and Salt Stresses Than Wild Type

**FIGURE 11:**
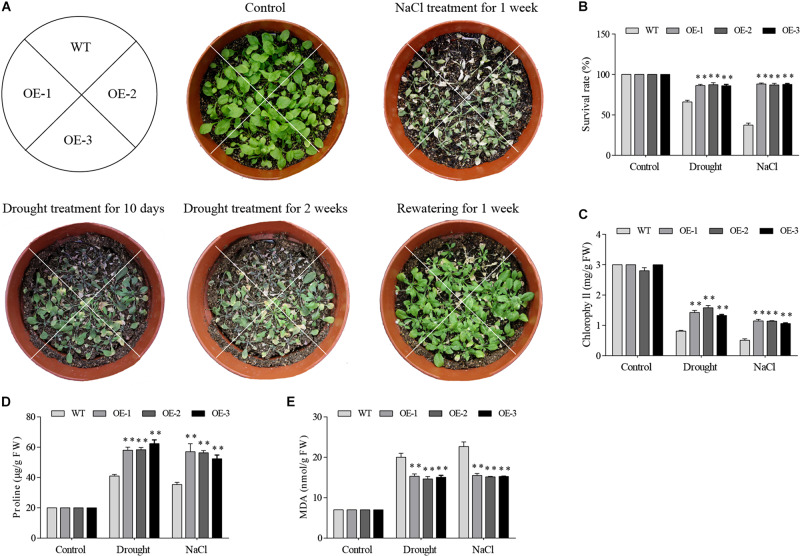
Overexpression of *GmGELP28* in *Arabidopsis* plants enhanced the tolerance to drought and salt stresses. **(A)** Phenotypes of three-week-old WT and transgenic plants under salt and drought stresses. **(B)** The survival rates of WT and transgenic plants upon rewatering after drought and NaCl treatments. **(C–E)** The leaf contents of chlorophyll, proline and MDA in WT and transgenic plants under normal and stress conditions, respectively. The data are shown as the means ± SD obtained from three biological replicates. ANOVA test demonstrates that there are significant differences (*p < 0.05, **p < 0.01).

Among 7 *GmGELP* genes, *GmGELP28* was selected to validate the role in abiotic stresses owing to significantly up-regulated expression levels by all tested treatments. Three independent *GmGELP28-*overexpression *Arabidopsis* lines (OE lines) were used to investigate the performance in drought and salt stresses. The results revealed that no visible differences were observed between WT lines and OE lines under normal growth conditions (Figure 11A). But after withholding water for 2 weeks, WT plants exhibited seriously hypersensitive symptoms to drought and NaCl treatments, and only approximately 67% and 38% survival rates were observed upon rewatering after drought and NaCl treatments, respectively (Figure 11B). The contents of MDA, proline and chlorophyll were measured under both normal and abiotic stress conditions. Compared with WT plants, OE lines showed significantly higher contents of chlorophyll and proline in their leaves (Figures 11C,D), and featured a significantly lower MDA content (Figure 11E).

### *GmGELP28* Improves Drought and Salt Stress Tolerance in Transgenic Soybean Hairy Roots

**FIGURE 12:**
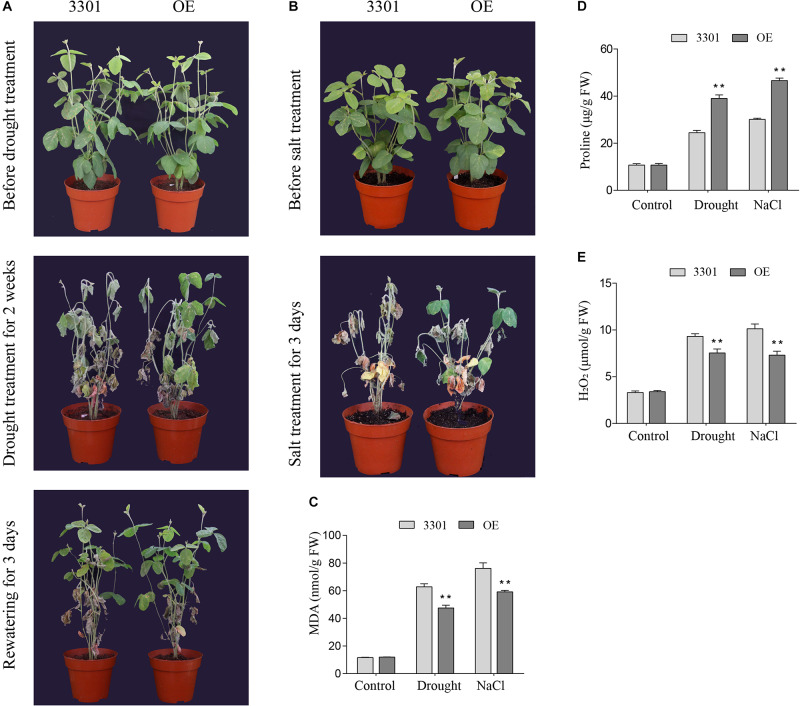
Overexpression of *GmGELP28* in soybean plants enhanced the tolerance to drought and salt stresses **(A,B)** Phenotypes of plants with transgenic soybean hairy roots subjected to drought and salt stresses, respectively. **(C–E)** The leaf contents of MDA, proline and H_2_O_2_ in empty vector control plants and transgenic plants under normal and stress conditions, respectively. The data are shown as the means ± SD obtained from three biological replicates. ANOVA test demonstrates that there are significant differences (*p < 0.05, **p < 0.01).

Similarly, the drought and salt tolerance tests were performed in *Agrobacterium rhizogenes*-mediated soybean hair roots. RT-qPCR analysis showed that the expression level of *GmGELP28* in transgenic plants was significantly higher than in empty vector control plants. For drought tolerance assays, 2-weeks soybean plants were subjected to drought stress. After 2 weeks of water deprivation, empty vector control plants exhibited severe water loss and significant withering relative to transgenic plants (Figure 12A). In addition, significantly more dried leaves were observed in empty vector control plants compared with transgenic plants after rewatering (Figure 12A). Under salt treatment, all empty vector control plants were died. In contrast, a large proportion of leaves in transgenic plants were green at this time point (Figure 12B). The contents of MDA, proline and H_2_O_2_ were important parameters involved with the tolerance to abiotic stresses. Under normal growth conditions, no significant differences in the contents of MDA, proline and H_2_O_2_ were observed. However, transgenic plants showed higher proline content and lower MDA and H_2_O_2_ contents than empty vector control plants when subjected to drought and salt treatments (Figures 12C–E).

## Discussion

GDSL esterase/lipase proteins (GELP) are a subfamily of lipolytic enzymes that have been discovered relatively recently (Chepyshko et al., 2012; Dong et al., 2016), and descriptions of the *GDSL* gene family and function have been identified in only a few plants (Dong et al., 2016; Cao et al., 2018). In fact, to the best of our knowledge, this is the first study to identify and characterize the soybean *GDSL* gene family. This paper set out to identify *GELP* members, and a search for *GELP* genes in soybean genome resulted in the identification of 194 members. The study aims to explore the phylogeny and evolution of the *GDSL* gene family, and to generate fresh insight into the different gene structures of *GmGELPs*. On the other hand, the study also systematically investigated the expression patterns of *GmGELP* members in different tissues and under drought, NaCl and ABA stress treatments.

A comparison of the number of *GELP* genes in soybean with several sequenced genomes displays that the *GDSL* gene family have a relatively large number (Chepyshko et al., 2012; Dong et al., 2016; Lai et al., 2017). Our study suggests the members of *GDSL* gene family are 1.86-times (194/104) more abundant in soybean than *Arabidopsis*. In accordance with the present results, a previous study demonstrated that the number of predicted protein-coding genes in the soybean genome was 70% higher than in *Arabidopsis* (Schmutz et al., 2010). Another significant feature of some GDSL lipases is their clustered distribution on chromosomes. In *Arabidopsis*, there were 16 cases of 2-9 genes arranged in tandem, in which one tandemly duplicated *AtGELP* gene sets contained 9 members (*AtGELP6* to *AtGELP14*) on chromosome 1 (Lai et al., 2017). In rice, approximately 47% *GELP* genes (54 out of 114) were closely arranged on chromosomes, comprising 17 clusters, in which closely linked genes were adjacent or isolated by 1-4 genes (Chepyshko et al., 2012). Genome-wide duplication is a large-scale process of gene multiplication at the chromosomal level, which has developed the architecture and function of many higher eukaryotic genomes, and leading to highly duplicated genomes with about 75% of genes existing in multiple copies (Schmutz et al., 2010; Lee et al., 2013). Genome-wide duplication also plays a vital role in the expansion of some families, which generates the largest number of duplicated genes. For example, the plant *MAPK*/*MAPKK* gene family was more likely to be expanded through whole genome or large segmental duplication events (Hamel et al., 2006). Consistent with this, 71% (137 out of 194) of *GDSL* family members occurred as WGD/segmental duplication within the soybean genome, which is the main driving force for the expansion of the soybean *GDSL* gene family.

Extensive loss and insertion of introns occurred during the evolution of eukaryotes, and gene duplication accelerated this process (Roy and Penny, 2007). However, the evolution of intron in duplicated genes seems to be of particular interest. For this purpose, a parsimonious reconstruction of the evolution for introns in subfamilies K and I was conducted to investigate the intron dynamics in duplicated genes on genome scale. Our results show a strong non-uniform distribution between subfamilies K and I. In addition, most intron positions are shared in duplicated genes, which reflects evolutionary conservation (Figure 7). In agreement with previous studies suggesting that the high conservation of intron position and phase was found more widely across angiosperms (Roy and Penny, 2007; Schmutz et al., 2010). The gene structure of more than 600 GDSL lipases from different plants had been analyzed, and the relative location of 6 conservative introns in the three subfamilies were found to be significantly different: intron positions 1 and 6 were present in all three subfamilies, intron position 5 was conserved in subfamilies A and B, while intron positions 2, 3 and 4 were specific to genes of subfamilies A, B and C, respectively (Volokita et al., 2011). However, the results of the current study do not support previous research. No conserved intron positions were found between subfamilies K and I, suggesting that the relative locations of the 13 introns in two subfamilies are significantly different, and further analysis reveal that the extra introns exist in only one or a few genes, which maybe generate by later events of intron gain (Figures 6, 7). Intron gain and loss have continually occurred in genome evolution at rather low rate, whereas the influences to structure divergence and functional differentiation are significant (Xu et al., 2012). Previous study show an excess of intron loss over intron gain in rice and *Arabidopsis* at low rate (Roy and Penny, 2007). A limitation of this study is that only 13 genes were used to analyze gene structure, from which the obtained conclusions cannot fully elucidate the evolutionary characteristics of introns. Therefore, there is abundant room for further progress in determining the mechanism of intron evolution.

It is well recognized that the expression of genes usually reflects their potential functions. The expression patterns of *GELP* genes in different tissues and abiotic stress conditions have been studied in several species, indicating the expression of a large number of *GELP* members can be induced by hormone, chemical, environmental stress as well as pathogen infection (Naranjo et al., 2006; Hong et al., 2008; Kim et al., 2008, 2014; Kwon et al., 2009; Chepyshko et al., 2012; Huang et al., 2015). For example, in rice, some *GELP* genes showed constitutive expression or nearly constitutive expression, with relatively high transcription levels in most tissues and organs during vegetative growth phase, while very weak expression abundances were detected in some reproductive organs, such as anthers and seeds (Chepyshko et al., 2012). Our results matched those observed in earlier studies, some *GmGELP* genes had only background expression under normal conditions, yet they were notably induced by multiple abiotic stress treatments. For instance, *GmGELP8*, *17*, *28* and *74* were only slightly expressed in normal conditions, but significantly higher expression levels were observed after drought treatment. In addition, it was noted that *GmGELP28* was found to show a relatively high expression in three abiotic stress treatments. It is well-known that gene duplication is a main source of functional differentiation, which greatly increases functional diversity and improves the adaptability of species to the environment. Duplication events are followed by gene diversity and loss, and gene loss is the common fate of most duplicated genes (Schmutz et al., 2010), while functionally different duplicated genes are more likely to be preserved during evolution (Schlueter et al., 2007). Consistent with the find, our results suggest that duplicated genes exhibit diverse expression patterns (Figures 8, 9), which may account for the large number of duplicated genes in soybean *GDSL* gene family. Furthermore, previous study show that segmentally duplicated genes tend to maintain the same expression pattern, while tandemly duplicated genes can produce rapid expression differentiation (Ganko et al., 2007).

A new candidate, named as *GmGELP28*, was isolated from soybean and overexpressed in *Arabidopsis* and soybean, and a series of experiments were performed to explore its role in abiotic stress tolerance. Under drought stress, transgenic *Arabidopsis* plants showed higher survival rates and chlorophyll content with respect to WT plants (Figures 11B,C). Proline is implicated as the most common osmoprotectant in plants, it is involved in maintaining plant homeostasis in responses to stress conditions (Szekely et al., 2008). The higher accumulation of proline was found in *GmGELP28* transgenic *Arabidopsis* and soybean plants under drought and salt stress conditions, suggesting that proline may contribute to the tolerance of *GmGELP28* transgenic plants to drought and salt stresses (Figures 11D, 12D). High salt level can cause oxidative damage and hypertonic stress (Zhu, 2016), and the contents of MDA and H_2_O_2_ can reflect the degree of damage to plants. A great accumulation of MDA and H_2_O_2_ was detected in *Arabidopsis* WT plants and empty vector control soybean plants grown under drought and salt stress conditions (Figures 11E, 12C,E). In conclusion, the changes in the physiological parameters suggest the positive role of *GmGELP28* in stresses.

## Conclusion

In conclusion, we found 194 *GELP* genes in the soybean genome, and a comprehensive analysis was performed, including phylogenetic analysis, evolutionary features and expression profiles. Finally, we identified a candidate *GELP* gene *GmGELP28* involved in drought and salt tolerance. These results provide insights for better understanding the evolutionary mechanism of soybean *GELP* genes and important reference for genetic improvement of soybean.

## Data Availability Statement

The raw data supporting the conclusions of this article will be made available by the authors, without undue reservation.

## Author Contributions

H-GS contributed to the conception of the study. H-GS, Z-SX, and D-HM drafted and revised the manuscript. JC, Y-BZ, Y-XW, and MC contributed to data analysis. X-HZ, T-TW, W-LW, and Y-ZM conceived of and designed the experiments. All authors reviewed and approved the final manuscript.

## Conflict of Interest

The authors declare that the research was conducted in the absence of any commercial or financial relationships that could be construed as a potential conflict of interest.

## Supplementary Material

FIGURE S1 Distribution frequency of *GELP* genes on 20 soybean chromosomes.

FIGURE S2 Putative motifs of each soybean GELP protein by MEME.

FIGURE S3 Motif logos detected in GmGELP proteins via MEME analysis.

FIGURE S4 Intron numbers of *GELP* genes in soybean.

TABLE S1 Description of soybean *GELP* genes.

TABLE S2 Multiple sequence alignment among 8 reported AtGELP proteins and their homologous proteins in soybean.

TABLE S3 The Ka/Ks ratios of duplicated *GELP* gene pairs.

TABLE S4 FPKM values of different tissues of soybean *GELP* genes.

TABLE S5 FPKM values under drought treatment of soybean *GELP* genes.

TABLE S6 FPKM values under NaCl treatment of soybean *GELP* genes.

TABLE S7 FPKM values under ABA treatment of soybean *GELP* genes.

TABLE S8 Primers and sequences used in this study.

## Abbreviations

ABA: Abscisic acid
ABRE: ABA-responsive elements
CDS: Coding sequence length
FPKM: Fragments per kilobase of transcript per million mapped reads
FW: Fresh weight
GELP: GDSL-type esterase/lipase proteins
GSDS: Gene structure display server
HMM: Hidden Markov model
JA: Jasmonate
Ka: Nonsynonymous substitution rate
Ks: Synonymous substitution rate
LTR: Low-temperature responsive
MBS: MYB binding site
MDA: Malonaldehyde
MEJA: Methyl jasmonate
NCBI: National Center for Biotechnology Information
PF: Pfam
RT-qPCR: Real-time quantitative polymerase chain reaction
SA: Salicylic acid
WGD: Whole genome duplication.
