# Supplementary figures and images for: Genome-Wide Identification, Evolution, and Expression of GDSL-Type Esterase/Lipase Gene Family in Soybean
Source: Front Plant Sci. 2020 Jun 25;11:726. doi: 10.3389/fpls.2020.00726 (PMC7332888; doi:10.3389/fpls.2020.00726)

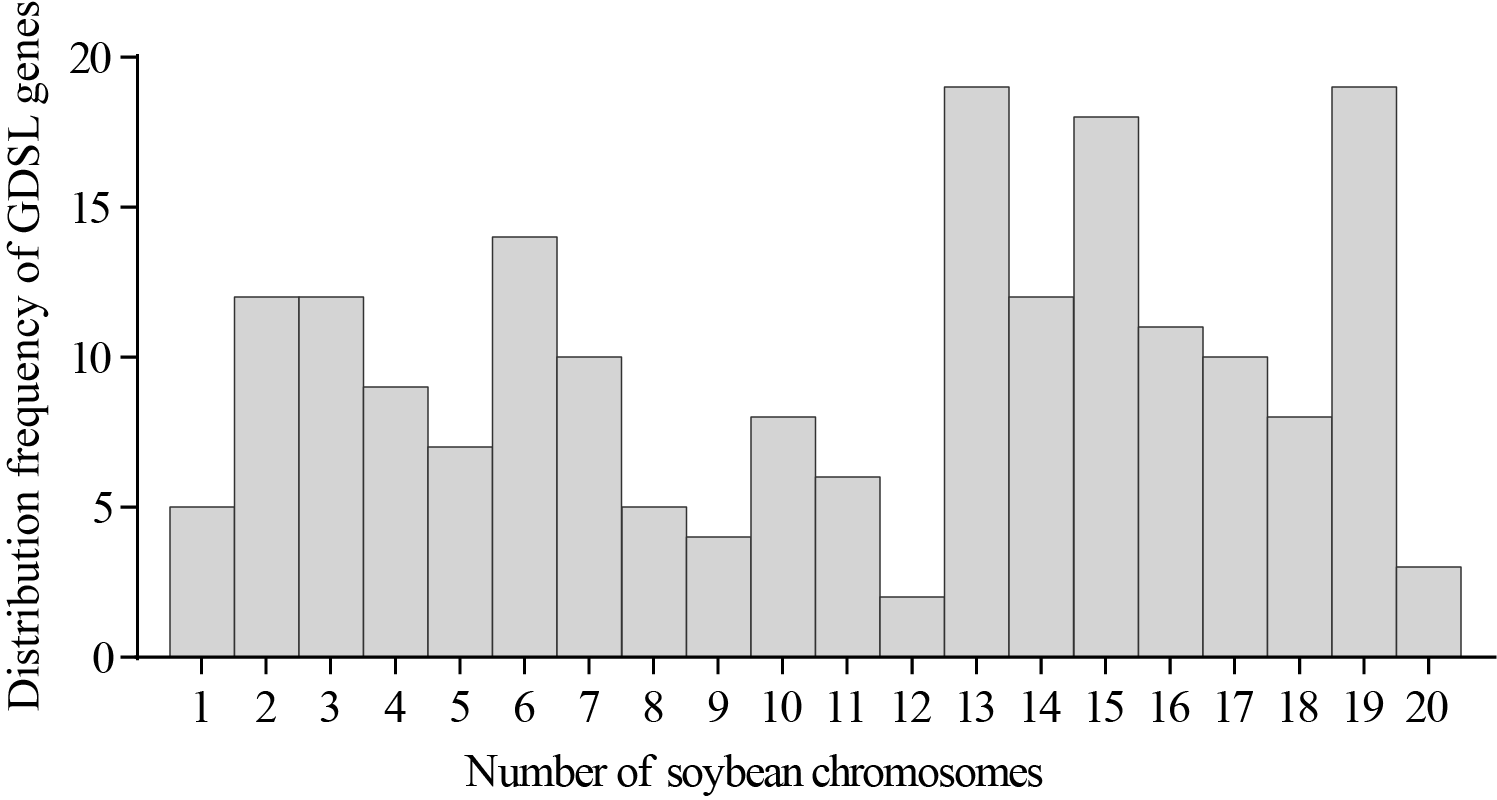

Supplement: FIGURE S1 — Distribution frequency of GELP genes on 20 soybean chromosomes. [file Image_1.TIF]

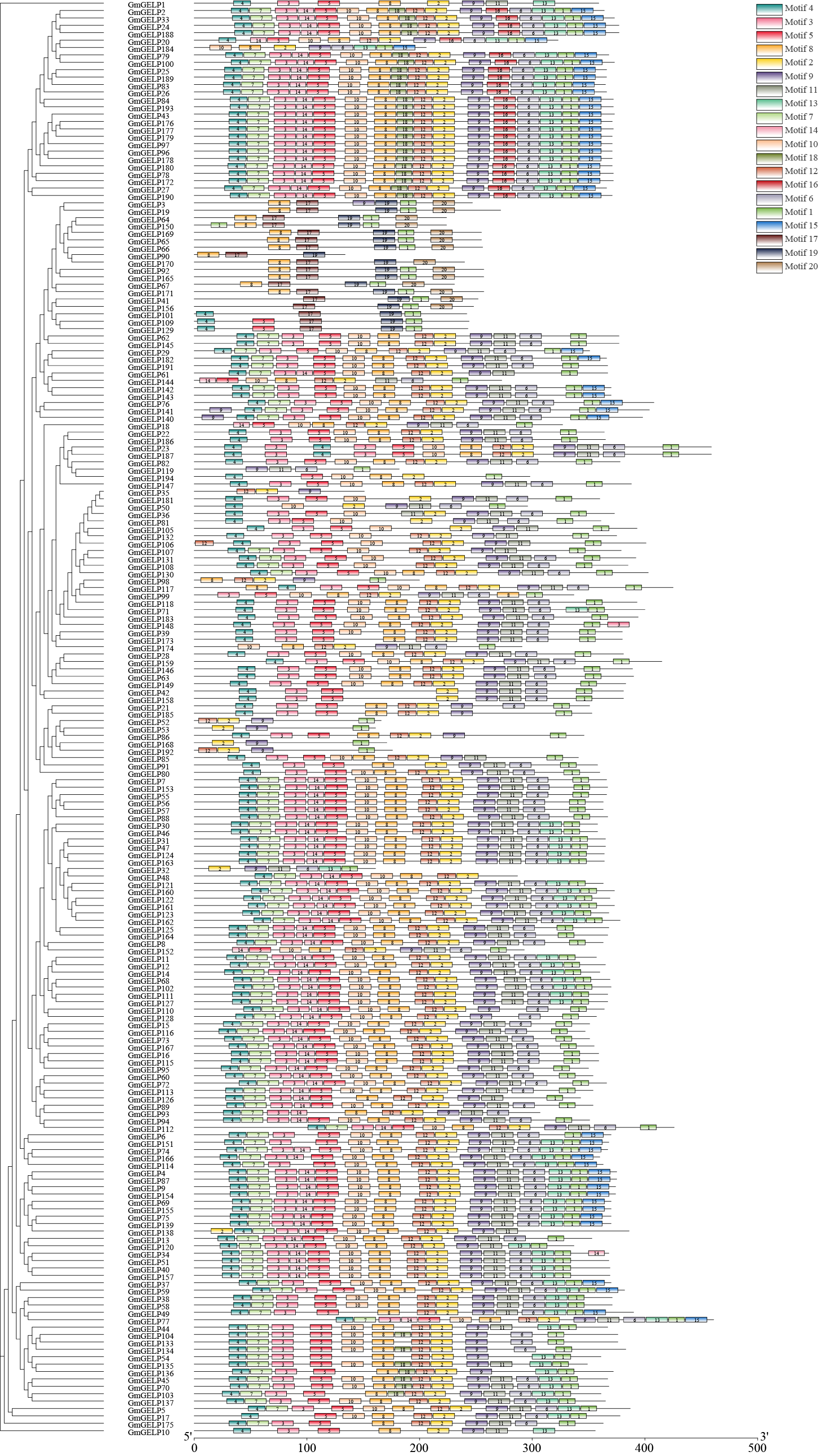

Supplement: FIGURE S2 — Putative motifs of each soybean GELP protein by MEME. [file Image_2.TIF]

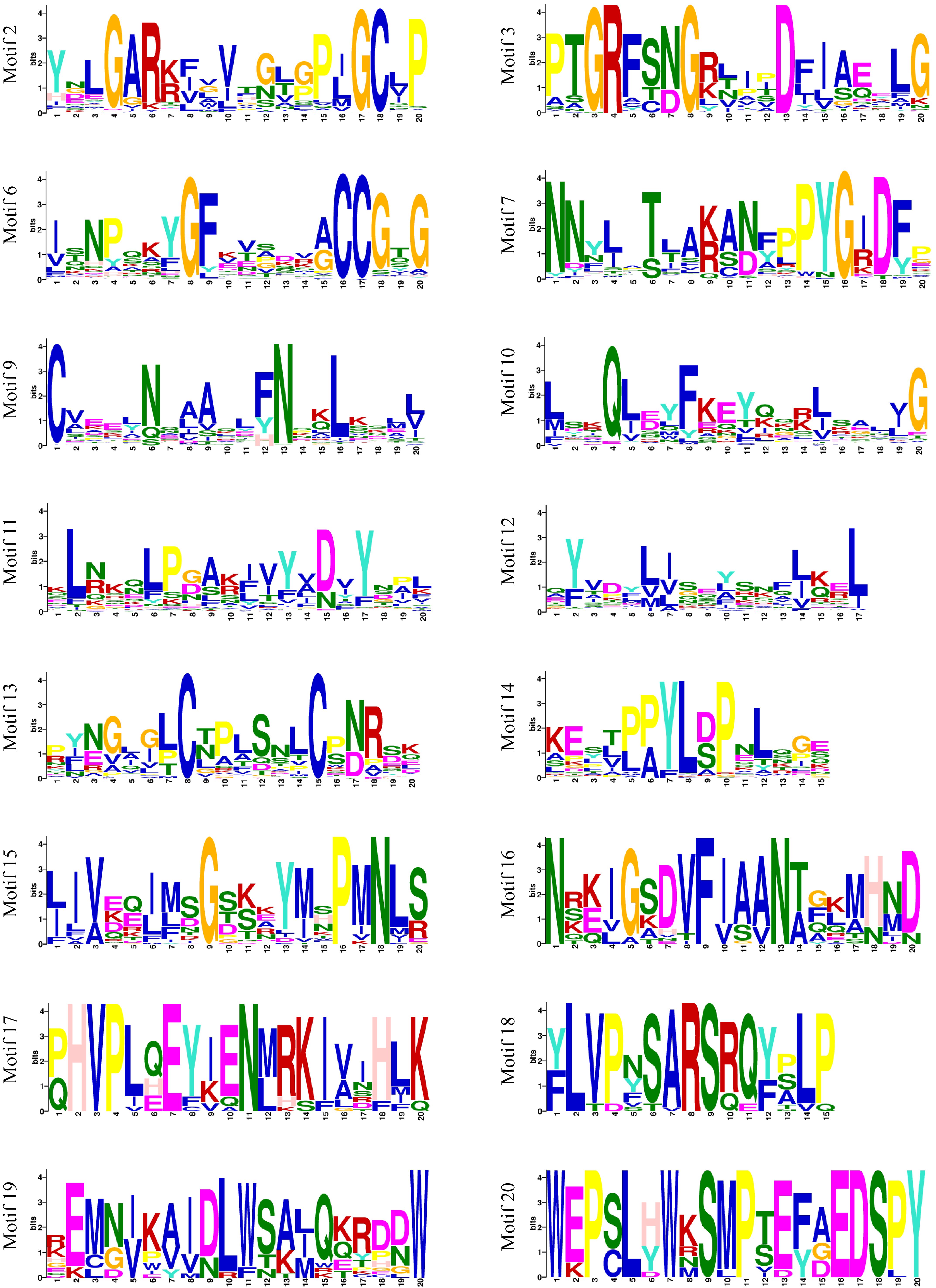

Supplement: FIGURE S3 — Motif logos detected in GmGELP proteins via MEME analysis. [file Image_3.TIF]

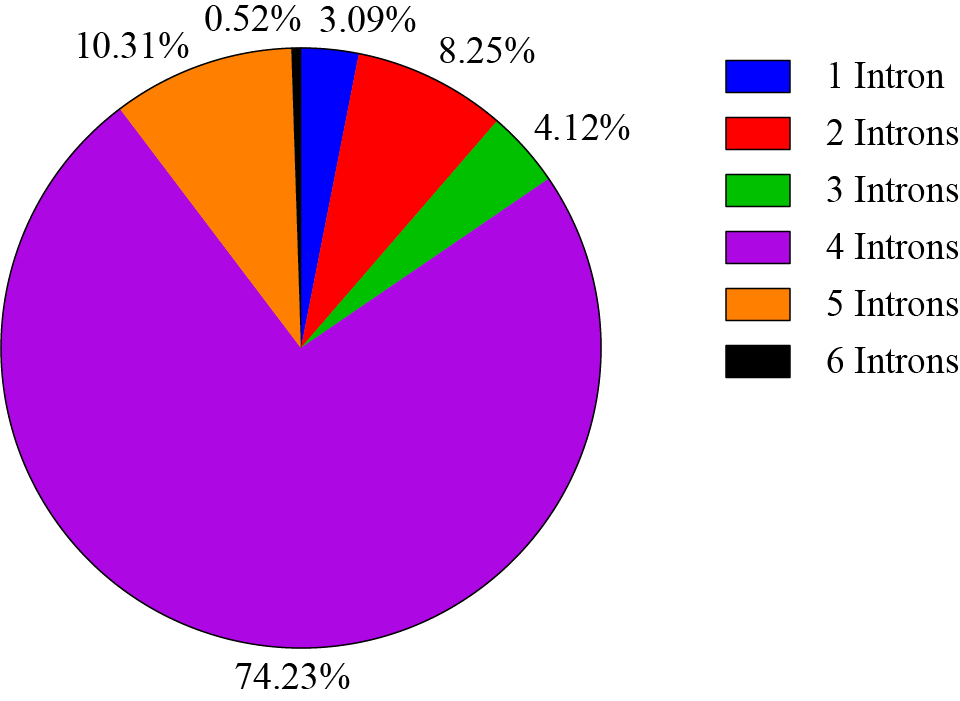

Supplement: FIGURE S4 — Intron numbers of GELP genes in soybean. [file Image_4.TIF]
